# Characterization of Oral *Enterobacteriaceae* Prevalence and Resistance Profile in Chronic Kidney Disease Patients Undergoing Peritoneal Dialysis

**DOI:** 10.3389/fmicb.2021.736685

**Published:** 2021-12-14

**Authors:** Carolina F. F. A. Costa, Ana Merino-Ribas, Catarina Ferreira, Carla Campos, Nádia Silva, Luciano Pereira, Andreia Garcia, Álvaro Azevedo, Raquel B. R. Mesquita, António O. S. S. Rangel, Célia M. Manaia, Benedita Sampaio-Maia

**Affiliations:** ^1^Instituto de Ciências Biomédicas Abel Salazar, Universidade do Porto, Porto, Portugal; ^2^Nephrology & Infectious Diseases R&D Group, INEB – Instituto de Engenharia Biomédica, i3S – Instituto de Investigação e Inovação em Saúde, Universidade do Porto, Porto, Portugal; ^3^Nephrology Department, Hospital Universitari Doctor Josep Trueta, Girona, Spain; ^4^Universidade Católica Portuguesa, CBQF - Centro de Biotecnologia e Química Fina – Laboratório Associado, Escola Superior de Biotecnologia, Porto, Portugal; ^5^Instituto Português de Oncologia do Porto Francisco Gentil (IPO), Porto, Portugal; ^6^Nephrology Department, Centro Hospitalar Universitário de São João, Porto, Portugal; ^7^Faculdade de Medicina Dentária, Universidade do Porto, Porto, Portugal; ^8^Laboratório para a Investigação Integrativa e Translacional em Saúde Populacional (ITR), Porto, Portugal

**Keywords:** chronic kidney disease, oral microbiome, oral dysbiosis, peritoneal dialysis, *Enterobacteriaceae*, *Raoultella ornithinolytica*, antibiotic resistance

## Abstract

Chronic Kidney Disease (CKD) is a growing public-health concern worldwide. Patients exhibit compromised immunity and are more prone to infection than other populations. Therefore, oral colonization by clinically relevant members of the *Enterobacteriaceae* family, major agents of both nosocomial and dialysis-associated infections with frequent prevalence of antibiotic resistances, may constitute a serious risk. Thus, this study aimed to assess the occurrence of clinically relevant enterobacteria and their antibiotic resistance profiles in the oral cavity of CKD patients undergoing peritoneal dialysis (CKD-PD) and compare it to healthy controls. Saliva samples from all the participants were cultured on MacConkey Agar and evaluated regarding the levels of urea, ammonia, and pH. Bacterial isolates were identified and characterized for antibiotic resistance phenotype and genotype. The results showed that CKD-PD patients exhibited significantly higher salivary pH, urea, and ammonia levels than controls, that was accompanied by higher prevalence and diversity of oral enterobacteria. Out of all the species isolated, only the prevalence of *Raoultella ornithinolytica* varied significantly between groups, colonizing the oral cavity of approximately 30% of CKD-PD patients while absent from controls. Antibiotic resistance phenotyping revealed mostly putative intrinsic resistance phenotypes (to amoxicillin, ticarcillin, and cephalothin), and resistance to sulfamethoxazole (~43% of isolates) and streptomycin (~17%). However, all isolates were resistant to at least one of the antibiotics tested and multidrug resistance isolates were only found in CKD-PD group (31,6%). Mobile genetic elements and resistance genes were detected in isolates of the species *Raoultella ornithinolytica*, *Klebsiella pneumoniae*, *Klebsiella oxytoca*, *Escherichia coli*, and *Enterobacter asburiae*, mostly originated from CKD-PD patients. PD-related infection history revealed that *Enterobacteriaceae* were responsible for ~8% of peritonitis and ~ 16% of exit-site infections episodes in CKD-PD patients, although no association was found to oral enterobacteria colonization at the time of sampling. The results suggest that the CKD-induced alterations of the oral milieu might promote a dysbiosis of the commensal oral microbiome, namely the proliferation of clinically relevant *Enterobacteriaceae* potentially harboring acquired antibiotic resistance genes. This study highlights the importance of the oral cavity as a reservoir for pathobionts and antibiotic resistances in CKD patients undergoing peritoneal dialysis.

## Introduction

Chronic kidney disease (CKD) is an increasing global health issue, defined as decreased kidney function shown by a glomerular filtration rate of less than 60 ml/min/1.73 m^2^, or markers of kidney damage, or both, for a duration of at least 3 months (Webster et al., 2017; Benjamin and Lappin, 2021). CKD is commonly progressive and when patients reach end-stage renal disease, they may require renal replacement therapies, such as hemodialysis, peritoneal dialysis (PD) or kidney transplantation (Murdeshwar and Anjum, 2021). CKD patients exhibit increased mortality and morbidity, especially associated with cardiovascular events (Webster et al., 2017; Benjamin and Lappin, 2021).

The decrease in kidney function leads to the accumulation of substances in the body that in normal conditions would be eliminated in the urine (Lisowska-Myjak, 2014). These substances, known as uremic toxins, build up inside the organism and cause alterations in various body sites, including the oral cavity, with higher levels of urea, ammonia and pH being commonly detected in the saliva of CKD patients (Gupta et al., 2015; Simões-Silva et al., 2017). These changes are thought to be major selective factors responsible for altering the oral microbiome, which has shown significant association with CKD status (Simões-Silva et al., 2017, 2018; Hu et al., 2018).

On the other hand, it has been hypothesized that the oral microbiome may contribute in some extent to the progression of CKD. Three mechanisms have been proposed that link oral infection, especially periodontitis, to secondary systemic effects (Li et al., 2000; Simões-Silva et al., 2018). These include the generalized spread of infection from the oral cavity as a result of transient bacteremia; generalized injury resulting from circulating toxins of oral microbial origin; and systemic inflammation as a result of immunological activation induced by oral microorganisms (Li et al., 2000; Simões-Silva et al., 2018).

Among other complications, the accumulation of uremic toxins in the body leads to a chronic inflammatory status which can cause the suppression of lymphocytes and cell-mediated immunity, making CKD patients more prone to infections than other populations (Gupta et al., 2015). In fact, infectious episodes persist as the main weakness of PD programs, with peritonitis and exit-site/tunnel infections remaining as the most common and relevant concerns for these patients (Mujais, 2006; Liakopoulos et al., 2017). While Gram-positive agents (mainly *Staphylococcus* spp. and *Streptococcus* spp.) are responsible for the majority of infections, Gram-negative bacteria (mainly *Pseudomonas* spp. and members of the family *Enterobacteriaceae*) are more likely to provoke more severe infections with poor outcomes (Li and Chow, 2011; Liakopoulos et al., 2017).

The *Enterobacteriaceae* family is of particular relevance in end-stage renal disease patients. Aside from being recognized agents of nosocomial infections, *Enterobacteriaceae* are responsible for 10 to 12% of all peritoneal dialysis-associated peritonitis, constituting a significant risk for patients on PD programs (Jain and Blake, 2006; Szeto et al., 2006). Regarding the oral microbiome, enterobacteria are frequent inhabitants of periodontal pockets (Gonçalves et al., 2007), potentially contributing to generalized inflammation and disease progression in these patients. Furthermore, *Enterobacteriaceae* are critical level pathogens regarding antibiotic resistance phenotypes. The mechanisms of antibiotic resistance in these bacteria may be intrinsic due to the presence of features encoded by chromosomal genes related with functions such as antibiotic inactivating enzymes and non-enzymatic paths (efflux pumps, permeability or target modifications) that are observed in all or most of the members of a given bacterial species (Ruppé et al., 2015). In contrast, acquired antibiotic resistance is due to gene mutation or, more frequently, to incorporation of genes received through mobile genetic elements, such as plasmids or phages that often harbor genes encoding ß-lactamases, aminoglycosides modifying enzymes, or non-enzymatic mechanisms (Cox and Wright, 2013).

Antimicrobial resistant enterobacteria are quite prevalent in CKD patients and are the cause of serious drug-resistant infectious episodes (Wang et al., 2019). Probably because of frequent hospitalizations and frequent need for antibiotic therapy, patients with CKD tend to have more infections by drug resistant bacteria than people with normal kidney function (Fysaraki et al., 2013; Salloum et al., 2020). In 2014, outpatient hemodialysis facilities in the United States reported that, out of the isolates identified as agents of bloodstream infections and exit-site-related infections, 17.8% of *Escherichia coli* and 14.6% of *Klebsiella* spp. isolates were resistant to cephalosporins and 4.8% of *Enterobacter* spp. isolates were resistant to carbapenems (Nguyen et al., 2017). In another study conducted in a hemodialysis unit in a hospital in Algeria, 100% of *K. pneumoniae* isolates associated with catheter-related infections produced extended spectrum beta-lactamase and were resistant to at least gentamicin (Sahli et al., 2017). Regarding peritoneal dialysis, in PD patients who developed dialysis-associated peritonitis between 2002 and 2011, 54.3% of Enterobacteriaceae isolates were resistant to third-generation cephalosporins and 76.1% were resistant to fluoroquinolones (ciprofloxacin), and all of them produced extended spectrum beta-lactamase (Prasad et al., 2014). This data shows that a large percentage of enterobacteria responsible for dialysis-related infections in CKD patients also exhibit resistance to one or more antibiotic drugs.

Taking into consideration the above mentioned, this work aimed to assess the prevalence and antibiotic resistance profile of clinically relevant enterobacteria in the oral cavity of CKD patients undergoing PD.

## Materials and Methods

### Participant Recruitment

Chronic kidney disease patients undergoing peritoneal dialysis (CKD-PD, *n* = 44) followed at the outpatient clinic of the Nephrology Department of Centro Hospitalar Universitário de São João were invited to participate in this study. Exclusion criteria for this group included inability to give informed consent, pregnancy, recent history of infection (less than 3 months), recent history of antibiotic therapy (less than 3 months), and severe acute illness. Samples were collected according to patient attendance to the clinic over a period of 4 months. Relevant clinical and demographic information was gathered for each individual through a semi-structured interview and by reviewing the patient’s computerized clinical reports. The occurrence of PD-related infectious episodes associated with species from the *Enterobacteriaceae* family was retrieved from clinical records from the date of entrance in the PD program until the moment of oral sample collection.

The control group was recruited from the student body of the Faculdade de Medicina Dentária da Universidade do Porto (*n* = 37). Relevant clinical and demographic information was gathered through a semi-structured interview. Exclusion criteria for the control group included inability to give informed consent, pregnancy, recent history of infection (less than 3 months), recent history of antibiotic therapy (less than 3 months), and compromised oral health (occurrence of gum disease, such as gingivitis or periodontitis, and a caries index including decayed, missing or filled teeth superior to 5). Controls had no history of chronic or systemic diseases (such as diabetes, hypertension, obesity, allergies, autoimmune diseases, etc.), no history of genetic disorders, no history of renal disease, and had not been diagnosed for any other health condition.

The protocols for the study were approved by both the Ethics Committee for Health of Centro Hospitalar Universitário de São João (approval number 200/18) and the Ethics Committee for Health of Faculdade de Medicina Dentária da Universidade do Porto (approval number 12/2019) and followed the 1964 Helsinki declaration and its later amendments; all recruited participants gave their written informed consent.

### Sample Collection

Unstimulated whole saliva was collected *via* the spitting method (Navazesh, 1993) into sterile containers, at least 1 h after eating or tooth brushing. Prior to the collection, the subjects did a water mouthwash to minimize typical dry-mouth sensation and to clean the oral cavity. Saliva collection began after swallowing the residual saliva present in the mouth and allowing newly produced saliva to accumulate. Part of the saliva samples (~1 ml) were stored at −20°C until biochemical analysis and the remaining volume was kept refrigerated for microbiological evaluation.

### Saliva Biochemical Analysis

The biochemical analysis was carried out using a sequential injection analysis system with potentiometric detection for the determination of urea and ammonia in saliva samples as previously described (Thepchuay et al., 2020), and the pH was measured using pH strips (5.0–8.0, Duotest, Germany and 1–14, Merck, Germany).

### Oral Microbiota Isolation and Identification

Up to 2 h after collection, refrigerated saliva samples were serially diluted with sterile 0.9% NaCl solution and were plated in triplicate in MacConkey Agar (VWR Chemicals BDH, Belgium). This culture medium was used in order to target non-fastidious non-strict anaerobic Gram-negative species. After aerobic incubation at 37°C for 48 h, the total number of colonies was counted and the results were expressed in colony-forming units per mL of saliva (CFU/ml).

From each saliva sample, all colonies with distinct appearances were reisolated in the same medium, which resulted in the selection of 2 to 3 isolates per individual saliva sample. Additionally, urease production was tested by incubating colonies on Urea Broth (Panreac, Spain) at 37°C for 24 h. Isolates were then stored in Brain Heart Infusion broth (BHI; Biolab Zrt., Hungary) supplemented with 10% glycerol at −80°C and were later identified by matrix-assisted laser desorption/ionization–time-of-flight mass spectrometry (MALDI-TOF MS, Bruker MALDI Biotyper).

The MALDI-TOF MS analysis was conducted directly from fresh (<24 h) bacterial colonies according to manufacturer’s instructions. When necessary, formic acid 70% was added to samples to improve identification. Only isolates which obtained an identification score value equal or higher than 2.0, corresponding to probable identification to species level, were considered properly identified.

### Antibiotic Resistance Phenotype and Genotype

Antibiotic resistance phenotype was determined using the Kirby–Bauer disk-diffusion method to test the following antibiotics: amoxicillin (AML, 25 μg), gentamycin (GEN, 10 μg), ciprofloxacin (CIP, 5 μg), sulfamethoxazole/trimethoprim (SXT, 1.25/23.75 μg), tetracycline (TET, 30 μg), cephalothin (CP, 30 μg), meropenem (MEM, 10 μg), ceftazidime (CAZ, 30 μg), ticarcillin (TIC, 75 μg), colistin (CT, 50 μg), sulfamethoxazole (SUL, 25 μg) and streptomycin (STR, 10 μg; Oxoid^™^, Hampshire, UK). Assays were conducted as recommended for *Enterobacteriaceae* (CLSI, 2017). As a quality control, *Escherichia coli* strain ATCC 25922 was included in all assays.

PCR-based gene screening (primers and PCR conditions described in Supplementary Table S1) used DNA extracts obtained from fresh pure cultures: bacterial colonies were resuspended in 50 μl of nuclease-free water and heated for 10 min at 95°C; subsequently, the mixture was refrigerated on ice for 5 min and centrifuged at 14 000 rpm for 5 min for sedimentation of cell debris; the supernatant was used as template for PCR reactions (Wiedmann-al-Ahmad et al., 1994). PCR gel electrophoresis pictures are presented in Supplementary Figures S1–S9. The screened genetic determinants included resistance determinants to β-lactams (*bla*_TEM_, *bla*_CTX-M_, *bla*_OXA_ and *bla*_SHV_), sulfonamides (*sul1*) and quinolones (*qnrS*), and mobile genetic elements related with genetic recombination and gene acquisition, plasmid replicon types (incF, incHI1 and incHI2), representative of incompatibility groups circulating among *Enterobacteriaceae*, and class 1 integron integrase (*intI1*).

### Data Analysis

All data analysis was carried out using IBM SPSS Statistics for Windows, Version 23.0 (IBM Corp, NY, United States). When appropriate, the chi-square independence test was used to analyze hypotheses regarding the categorical variables, using continuity correction for 2×2 Tables. T-test for independent samples was used for the continuous variables, using Levene’s test for equal variances. In order to evaluate potential confounders like age and sex, binary logistic regression (Enter method) was applied for the presence of *Raoultella ornithinolytica* as dependent variable and the type of participant as another covariate with bootstrapping technique with 95% of confidence. A significance level of 0.05 was considered.

## Results

### Clinical Characterization of CKD-PD Patients

Clinical information relating to the CKD-PD patients is included in Table 1. The most prevalent etiology of CKD was diabetic nephropathy and, at the time of sampling, the average time on PD therapy was 32 months, ranging from 2 months up to 12.4 years. Regarding salivary biochemical parameters, pH, urea, and ammonia levels were all significantly higher in CKD-PD patients compared to healthy controls (Table 2).

**Table 1:** CKD-PD patients’ clinical information, including etiology of CKD, residual renal function, blood pressure, time on PD, and PD-related infection history.

	CKD-PD patients
Etiology of CKD (%)
Diabetic nephropathy	25.0%
Hepatorenal polycystic disease	20.5%
Glomerular disease	13.7%
Urologic (nephrectomy, neoplasia, chronic PNC)	9.1%
Chronic interstitial nephritis	4.5%
Nephroangiosclerosis	2.3%
Vasculitis	2.3%
Unknown	22.7%
Residual renal function (mL/min)	5.9 ± 4.0
Blood pressure (mmHg)
Systolic	140 ± 18
Diastolic	80 ± 10
PD vintage (months)	31.7 ± 30.2
Infection history^a^ (%)
Peritonitis	27.3%
By *Enterobacteriaceae*	2.3%
Exit-site infections	45.5%
By *Enterobacteriaceae*	9.1%

*Results are shown in prevalence (%) or mean ± SD. ^a^Prevalence of infections refers to PD patients that had at least one infection episode from the date of entrance in the PD program until the moment of oral sample collection. CKD, chronic kidney disease; PD, peritoneal dialysis; CKD-PD, chronic kidney disease patients undergoing peritoneal dialysis*.

**Table 2:** Demographic information and biochemical salivary parameters of healthy controls and CKD-PD patients.

	Controls	CKD-PD patients	*p* value
Age (years)	19.7 ± 1.2	55.9 ± 11.0	<0.001
Sex (male %)	18.9%	65.9%	<0.001
Saliva Biochemistry
pH	6.7 ± 0.3	8.1 ± 0.8	<0.001
Urea (mg/dL)	20.4 ± 14.2	85.2 ± 46.2	<0.001
Ammonia (mg/dL)	16.1 ± 12.8	63.6 ± 38.1	<0.001

*Results are shown in prevalence (%) or mean ± SD. CKD-PD, chronic kidney disease patients undergoing peritoneal dialysis*.

### Oral *Enterobacteriaceae* Prevalence

CKD-PD patients exhibited significantly higher microbial counts and *Enterobacteriaceae* prevalence (Table 3). *Enterobacteriaceae* diversity was also higher in CKD-PD patients, with only three species being isolated from healthy controls while seven species (two in common with the controls and an additional five) were isolated from the study group, from a total of eight isolated species (Table 3). Six of the total eight species tested positive for urease production (excluding *Escherichia coli* and *Klebsiella aerogenes*; Table 3). Out of all *Enterobacteriaceae* species identified, only *Raoultella ornithinolytica* exhibited significantly higher prevalence in CKD-PD patients, being absent from the oral cavity of healthy controls.

**Table 3:** Bacterial counts in MacConkey Agar and prevalence of participants colonized by urease producing (^*^) and non-producing *Enterobacteriaceae* species.

	Controls	CKD-PD patients	*P* value
Counts, CFU/ml	8.8 × 10^3^ ± 1.5 × 10^4^	3.1 × 10^4^ ± 4.9 × 10^4^	0.008
*Enterobacteriaceae* prevalence, %	10.8%	43.2%	0.003
*Raoultella ornithinolytica* ^*^	0%	29.5%	0.001
*Klebsiella oxytoca* ^*^	0%	2.3%	>0.999
*Klebsiella pneumoniae* ^*^	5.4%	2.3%	0.878
*Klebsiella aerogenes*	0%	2.3%	>0.999
*Enterobacter asburiae* ^*^	2.7%	4.5%	>0.999
*Escherichia coli*	2.7%	0%	0.930
*Citrobacter koseri* ^*^	0%	2.3%	>0.999
*Citrobacter freundii* ^*^	0%	2.3%	>0.999

Results are shown in prevalence (%) or mean ± SD. ^*^Species that tested positive for urease production. CKD-PD, chronic kidney disease patients undergoing peritoneal dialysis.

Because controls and CKD patients vary significantly in terms of age and gender, binary logistic regression was applied for the presence of *Raoultella ornithinolytica* as dependent variable containing three independent variables: sex, age and type of participants. The full model with bootstrapping technique containing all predictors was statically significant (*χ*^2^ (3, N = 81) = 18.041, *p* < 0.001). According to the bootstrapping technique, only the type of participants (controls or CKD) made a unique statistically significant contribution (*p* < 0.001) in the model (CKD patients’ coefficient, B = 20.299; CI95% = 17.675; 22.954). On the other hand, statistical significance was not attained for sex nor age, respectively (*p* = 0.760) and (*p* = 0.956).

### *Enterobacteriaceae*-Related Infection History

During the complete period of PD therapy, around one quarter of the studied population had at least one peritonitis and around half of the patients had at least one ESI (Table 1). When one looks to the total number of infectious episodes (Table 4), *Enterobacteriaceae* were responsible for 7.7% of all peritonitis and for 16.2% of all ESI episodes, and the identified infectious agents include the genera *Klebsiella*, *Enterobacter* and *Escherichia*. No association was found between previous *Enterobacteriaceae* infectious agents and the oral enterobacteria colonization of the CKD-PD patients at the time of sampling.

**Table 4:** Total number of PD-related infection episodes, including peritonitis and exit-site infections (ESI), and the infectious agent identified.

Infectious agent	PD-related infection episodes (n = 50)	
	Peritonitis (*n* = 13)	ESI (*n* = 37)
Others	12 (92.3%)	31 (83.8%)
Enterobacteria	1 (7.7%)	6 (16.2%)
*Klebsiella pneumoniae*	0	2 (5.4%)
*Klebsiella oxytoca*	1 (7.7%)	0
*Enterobacter* spp.	0	3 (8.1%)
*Escherichia coli*	0	1 (2.7%)

*PD, Peritoneal dialysis*.

### Antibiotic Resistance Profiling

*Enterobacteriaceae* isolates (*n* = 23) obtained from different individuals exhibited mainly intrinsic antibiotic resistance phenotypes (CLSI, 2017; EUCAST, 2020; Supplementary Table S2). Specifically, resistance to amoxicillin (in all isolates of *Klebsiella* spp., one isolate of *Citrobacter freundii*, 2 out of 3 isolates of *Enterobacter* spp., and 12 out of 14 isolates from *R. ornithinolytica*), resistance to ticarcillin (2 out of 3 isolates of *Klebsiella* spp. and in 10 out of 14 isolates from *R*. *ornithinolytica*), and resistance to cephalothin (in all isolates of *Enterobacter* spp.) were observed. In addition to intrinsic antibiotic resistance, sulfamethoxazole (in *Enterobacter asburiae*, *Escherichia coli*, *Klebsiella pneumoniae*, and *Raoultella ornithinolytica* isolates), and streptomycin (in *Enterobacter asburiae* and *Raoultella ornithinolytica* isolates) resistances were also observed. Only 6 isolates (2 isolates of *Enterobacter asburiae* and 4 of *Raoultella ornithinolytica*) exhibited multidrug resistance, all of them originated from CKD-PD patients. These results are depicted in Supplementary Table S2. Overall, out of the 23 *Enterobacteriaceae* isolates, about 83% were resistant to amoxicillin, 52% showed resistance to ticarcillin, 43% to sulfamethoxazole, 35% to ciprofloxacin and 17% to streptomycin. 100% of the isolates were resistant to at least one of the antibiotics tested. The percentage of sensitivity to the studied antibiotics is presented in Supplementary Figure S10.

Among the genes screened, neither the antibiotic resistance genes *bla*_TEM_, *bla*_OXA_ and *qnrS*, nor the plasmid replicon types incHI1 and incHI2 were detected. The other genetic determinants were observed in one *Escherichia coli* isolate from a healthy control (*sul1*); in 2 of the 3 *Enterobacter asburiae* isolates from CKD-PD patients and controls (*sul1*, *n* = 1 and *sul1* and *intI1*, *n* = 1); in the *Klebsiella oxytoca* isolate from a CKD-PD patient (*bla*_CTX-M_, normally associated with the plasmid replicon type incF); in 2 isolates of *Klebsiella pneumoniae* from controls (*bla*_SHV_, *n* = 2, probably intrinsic in this species, and *sul1*, *n* = 1); and in 4 of the 14 *Raoultella ornithinolytica* isolates from CKD-PD patients (*sul1* and *intI1*, *n* = 3 and *intI1*, *n* = 1). These results are depicted in Supplementary Table S2. Overall, the *bla*_SHV_ gene alone or with *sul1* and *bla*_CTX-M_ with replicon type incF were detected in 4.3% of isolates. The *sul1* gene alone was found in 8.7% of isolates and *sul1* with *intI1* were found in 17.4% of isolates. None of the tested genes were detected in approximately 57% of isolates. The prevalence of these genetic elements is presented in Supplementary Figure S11.

## Discussion

Overall, our results show that the accumulation of uremic toxins in patients with advanced CKD and the consequent changes in the oral milieu (such as the increase in salivary urea, ammonia and pH) may indeed act as selecting factors on the oral microbiome, leading to an oral dysbiosis with proliferation of pathobionts in the oral cavity. In fact, 6 of the 8 species identified in Table 3 tested positive for urease production. Thus, it was observed that more urease-producing species were found in the oral cavity of CKD patients in comparison to the oral cavity of healthy controls, which is expected to be due to the previously reported adaptation of the microbiome to the altered conditions of the oral milieu, evidenced in Table 2 (Hu et al., 2018; Rysz et al., 2021).

The increased prevalence of *Enterobacteriaceae* spp. is significantly relevant in CKD patients. While enterobacteria are only occasionally detected in the oral cavity of healthy individuals, colonization is much more prevalent in immunocompromised patients, people who come in contact with hospital environments frequently, and people suffering from hyposalivation, oral lesions, and systemic disease, a scenario that fits CKD patients (Peleg and Hooper, 2010; Posse et al., 2017). Perhaps, all these reasons also contributed to the higher prevalence of *Enterobacteriaceae* in the oral microbiota of our CKD patients as well as to the fact that *Enterobacteriaceae* were responsible for approximately 8% of the peritoneal dialysis-associated peritonitis and 16% of exit-site infections in our group of patients, highlighting the clinical relevance of our results.

The increased prevalence of *Raoultella ornithinolytica* in CKD patients undergoing PD is a particularly significant finding. Formerly classified as *Klebsiella ornithinolytica*, *R. ornithinolytica*, a histamine-producing aquatic-commensal enterobacteria, is a virulent pathogen of community-acquired and emerging nosocomial infections, particularly associated with invasive procedures (Seng et al., 2016; Hajjar et al., 2018, 2020). *R. ornithinolytica* is able to occasionally survive in human saliva and a case report has pointed it as a cause of primary peritonitis in humans (Sibanda, 2014; Seng et al., 2016), thus aggravating the risk of its presence in the oral cavity of patients undergoing peritoneal dialysis. Regarding the oral microbiome, the high rate of colonization by *Raoultella ornithinolytica* is a very interesting finding in itself, since this species is still not described in the Human Oral Microbiome Database.^1^

Although no association was found between previous *Enterobacteriaceae* infectious agents in the group of CKD patients and oral colonization by enterobacteria at the time of sampling, the difficulty in the identification of *R. ornithinolytica* and common misidentification as belonging to the *Klebsiella* genus should be considered, as a misidentification of the infectious agent is a possibility (Ponce-Alonso et al., 2016). Additionally, the hypothesis of an oral-peritoneal association regarding infection cannot be discarded without further studies, especially when all the *Enterobacteriaceae*-related infectious episodes reported in these patients occurred at least 1 year prior to the collection of the samples used in this study.

It is important to note that all the species identified in this study are clinically relevant and constitute potential cause of infection, with many being associated with PD-related infections (Barraclough et al., 2009; Li and Chow, 2011). Although contamination concerns for patients undergoing peritoneal dialysis typically tend to focus on contamination from external origins, the oral microbiome may be a major source of contamination both through external and internal routes (Li and Chow, 2011; Smith and Nehring, 2021). Bacteria inhabiting the oral cavity can translocate into the bloodstream as a result from routine daily activities, such as tooth brushing or invasive dental procedures, becoming opportunistic infectious agents in distant body sites such as the peritoneum (Smith and Nehring, 2021). In fact, the risk associated with the presence of pathogenic bacteria in the oral-nasal cavities of CKD patients is already recognized, which is why these patients are frequently screened for nasal carriage of *Staphylococcus aureus* as a way to prevent infection and the efficacy of prophylactic intranasal antibiotics for the treatment of confirmed nasal carriage of *S. aureus* has been tested in several prospective studies (Grothe et al., 2014; Li et al., 2016). Furthermore, the presence of these bacteria in the oral cavity can stimulate the immune system and contribute to the inflammatory state in CKD, which then leads to cardiovascular events, the main cause of death in these patients (Li et al., 2000; Webster et al., 2017; Höfer et al., 2021).

The oral cavity has also recently been highlighted as a potential reservoir of antibiotic resistance genes (Roberts and Mullany, 2010; Jiang et al., 2018; de Sousa Moreira Almeida et al., 2020). Resistance against amoxicillin was observed in 83% of isolates, although in these cases it is possibly intrinsic (Cox and Wright, 2013). However, putative acquired genetic elements related with antibiotic resistance were observed in 10 out of 23 oral isolates, 3 from healthy individuals (*sul1* in *Escherichia coli* and *bla*_SHV_ in *Klebsiella pneumoniae*) and 7 from CKD patients (*sul1*, *intI1*, *bla*_CTX-M_ and incF in *Enterobacter asburiae*, *Raoultella ornithinolytica* and *Klebsiella oxytoca*). Curiously, the 5 isolates harboring the gene *intI1* and the one exhibiting the high mobile replicon type incF and *bla*_CTX-M_ genes all originated from CKD patients. These observations raise the interest to investigate the vulnerability of some population groups to be colonized by bacteria harboring acquired antibiotic resistance genes and mobile genetic elements (de Sousa Moreira Almeida et al., 2020). Overall, regarding the resistance phenotype, 100% of the *Enterobacteriaceae* isolates were resistant to at least one of the antibiotics tested and only 6 isolates, all originating from CKD-PD patients, exhibited multidrug resistance. Genetic elements related to resistance were also detected in approximately 43% of isolates. Antibiotic-resistant enterobacteria are known agents of dialysis-associated infections in CKD patients, frequently originating serious life-threatening complications (Wang et al., 2019). Therefore, it is important to keep screening isolates from CKD patients for antimicrobial resistances, in order to plan efficient therapeutic interventions in case of infection.

Our study presents several strengths and some limitations. The fact that the control and CKD groups differ in age and gender may constitute a limitation of our study, although a binary logistic regression was applied using the prevalence of *Raoultella ornithinolytica* (the only member of the *Enterobacteriaceae* family with a significantly different prevalence between groups) as the dependent variable and sex, age, and type of participants as independent variables, confirming that only the type of participants (controls or CKD-PD) made a unique statistically significant contribution. Moreover, though ideally the groups should display similar demographic characteristics, several studies have reported that the oral microbiome does not suffer significant alterations throughout adulthood, with no significant differences being reported by gender or age groups (adults and young adults) at species level. Alterations in the healthy oral microbiota are only significant before adulthood or after the age of 65–70 years-old (Crielaard et al., 2011; Belstrøm et al., 2014; Lira-Junior et al., 2018; Verma et al., 2021). As so, the healthy young adults constituting the control group in this study are nevertheless representative of healthy reference values. Regarding methodology, although MALDI-TOF MS is a universally used method that has been routinely used in clinical microbiology (He et al., 2010; Tsuchida et al., 2020) and is considered extremely adequate for the identification of enterobacteria (Richter et al., 2013), it presents some limitations, as is the example of the inability to differentiate *Escherichia coli* from *Shigella* species (Devanga Ragupathi et al., 2017).

When one looks to the study strengths, firstly, it highlights how the uremic state in CKD can cause a dysbiosis of the oral microbiome and lead to a proliferation of enterobacteria, which are clinically relevant pathogens for patients on PD programs. Additionally, the identification of *Raoultella ornithinolytica* as a colonizer of the oral cavity is of extreme importance. The isolation of *R. ornithinolytica* from saliva samples has been reported before in the literature (Heggendorn et al., 2013; Derafshi et al., 2017), but never in such a high prevalence as in this study and never as an oral colonizer of CKD patients. It is important to keep in mind that this is an emerging species both in terms of infectious episodes in humans and in terms of resistance to antibiotic agents (Seng et al., 2016; Hajjar et al., 2020), two factors that make *R. ornithinolytica* a clinically relevant cause of concern, not only to CKD patients, but to the general population. The profiling of *Enterobacteriaceae* isolates in terms of antibiotic resistance, both regarding resistance phenotypes and the presence of genetic elements, is another strong point of this paper. As mentioned before, antibiotic-resistant enterobacteria are responsible for a significant percentage of infections in CKD patients (Prasad et al., 2014; Nguyen et al., 2017; Sahli et al., 2017; Wang et al., 2019), which makes their screening crucial for the clinical follow-up of said patients, given it can provide information for efficient antibiotic prescription in case of infection. Of note that infections are a major problem in peritoneal dialysis programs, leading to patients’ deaths or exclusion from peritoneal dialysis programs (Li et al., 2016). Therefore, the search of predisposing factors that lead to peritonitis and catheter-related infections in CKD-PD patients is of outmost importance. Finally, our study also contributes to the profiling of antibiotic resistance genes in the oral microbiome. In recent years, the oral cavity has been highlighted as a reservoir for resistance genes (de Sousa Moreira Almeida et al., 2020) and the profiling of these genes provides extremely relevant information for dentistry and the treatment of oral infections.

## Conclusion

Our results suggest that the accumulation of uremic toxins in CKD patients induces alterations in the oral milieu, which, in turn, exert selective pressure on the oral microbiome, leading to its dysbiosis with significantly increased prevalence of urease-producing enterobacteria, specifically of *Raoultella ornithinolytica*, an opportunistic enterobacteria. Antibiotic resistance phenotyping revealed mostly intrinsic resistances, although resistances to sulfamethoxazole and streptomycin were also detected. Multidrug resistance was exclusively found in CKD-PD patients isolates. These results, together with the detection of acquired antibiotic resistance genes, alert to the possibility of the oral microbiota as a potential reservoir of resistance genes.

*Enterobacteriaceae* are known opportunistic pathogens of CKD patients, and this CKD-induced oral dysbiosis can potentially lead to an increase of resistant infectious and inflammatory episodes, contributing, in turn, to the progression of the disease and to its mortality rate. For all these reasons, studying the oral microbiome and its associated resistome and comprehend its role in infection episodes and in the progression of systemic diseases is of extreme relevance to ensure the health and well-being of CKD patients undergoing peritoneal dialysis.

## Data Availability Statement

The raw data supporting the conclusions of this article will be made available by the authors, without undue reservation.

## Ethics Statement

The studies involving human participants were reviewed and approved by Ethics Committee for Health of Centro Hospitalar Universitário de São João: (approval number 200/18) and Ethics Committee for Health of Faculdade de Medicina Dentária da Universidade do Porto: (approval number 12/2019). The patients/participants provided their written informed consent to participate in this study.

## Author Contributions

RM, AR, and BS-M contributed to conception and design of the study. CC, AM-R, CC, NS, LP, CF and AG contributed to data acquisition. CC, AM-R, CC, CF, RM, AR, CM and BS-M contributed to data analysis and interpretation. CC, CF, AA, RM, AR, CM and BS-M performed the statistical analysis. CC wrote the first draft of the manuscript. All authors contributed to manuscript revision, read, and approved the submitted version.

## Funding

This work is a result of the project POCI-01-0145-FEDER-029777, co-financed by Competitiveness and Internationalisation Operational Programme (POCI), under the PORTUGAL 2020 Partnership Agreement, through the European Regional Development Fund (ERDF) and through national funds by the FCT – Fundação para a Ciência e a Tecnologia. CC fellowship was supported by FCT/MCTES scholarship with the reference 2020.08540.BD. This work and CF were financially supported by FEDER through project “Assessing the risks associated with environmental antibiotic resistant bacteria: propagation and transmission to humans” (PTDC/CTA-AMB/28196/2017) – Programa Operacional Competitividade e Internacionalização, and by National Funds from FCT – Fundação para a Ciência e a Tecnologia and was hosted by CBQF through FCT project UIDB/50016/2020.

## Conflict of Interest

The authors declare that the research was conducted in the absence of any commercial or financial relationships that could be construed as a potential conflict of interest.

## Publisher’s Note

All claims expressed in this article are solely those of the authors and do not necessarily represent those of their affiliated organizations, or those of the publisher, the editors and the reviewers. Any product that may be evaluated in this article, or claim that may be made by its manufacturer, is not guaranteed or endorsed by the publisher.

## Footnotes

^1^ http://www.homd.org/
