# Supplementary material for: Characterization of Oral Enterobacteriaceae Prevalence and Resistance Profile in Chronic Kidney Disease Patients Undergoing Peritoneal Dialysis
Source: Front Microbiol. 2021 Dec 14;12:736685. doi: 10.3389/fmicb.2021.736685 (PMC8713742; doi:10.3389/fmicb.2021.736685)
Supplement: Supplementary file 1 [file Table_1.DOCX]

Supplementary Material

# Supplementary Tables

**Supplementary Table 1.** PCR conditions used in this paper.

| **Description** | **Target** | **Primer** | **Sequence (5´to 3´)** | **Anealing temperature (°C)** | **Amplicon  Size (bp)** | **References** |
| --- | --- | --- | --- | --- | --- | --- |
| Beta-lactamase | *bla*_CTX-M_ | blaCTX-M (fw) | CRATGTGCAGYACCAGTAA | 53 | 540 | Weill et al., 2004 |
|  |  | blaCTX-M (rev) | CGCRATATCRTTGGTGGTG |  |  |  |
|  | *bla*_OXA-A_ | blaOXA-A (fw) | ACACAATACATATCAACTTCGC | 53 | 814 | Henriques et al., 2006 |
|  |  | blaOXA-A (rev) | AGTGTGTTTAGAATGGTGATC |  |  |  |
|  | *bla*_SHV_ | blaSHV (fw) | TCGGGCCGCGTAGGCATGAT | 62 | 626 | DiPersio et al., 2005 |
|  |  | blaSHV (rev) | AGCAGGGCGACAATCCCGCG |  |  |  |
|  | *bla*_TEM_ | blaTEM (fw) | ATAAAATTCTTGAAGACGAAA | 45 | 1080 | DiPersio et al., 2005 |
|  |  | blaTEM (rev) | GACAGTTACCAATGCTTAATCA |  |  |  |
| Sulfonamides | *Sul1* | sul1-F | TGGTGACGGTGTTCGGCATTC | 59 | 789 | Reis et al., 2018 |
|  |  | sul1-R | GCGAGGGTTTCCGAGAAGGTG |  |  |  |
| Quinolones | *qnrS* | qnrSm F | GCAAGTTCATTGAACAGGGT | 428 | 54 | Cattoir et al., 2007 |
|  |  | qnrSm R | TCTAAACCGTCGAGTTCGGCG |  |  |  |
| Class 1 integrase | *IntI1* | IntI1_F | CCT CCC GCA CGA TGA TC | 280 | 55 | Henriques et al., 2006 |
|  |  | IntI1_R | TCC ACG CAT CGT CAG GC |  |  |  |
| Plasmid replicons | F | FrepB FW | TGATCGTTTAAGGAATTTTG | 270 | 52 | Caratolli et al., 2005 |
|  |  | FrepB RV | GAAGATCAGTCACACCATCC |  |  |  |
|  | HI1 | HI1 FW | GGAGCGATGGATTACTTCAGTAC | 471 | 60 |  |
|  |  | HI1 RV | TGCCGTTTCACCTCGTGAGTA |  |  |  |
|  | HI2 | HI2 FW | TTTCTCCTGAGTCACCTGTTAACAC | 644 | 60 |  |
|  |  | HI2 RV | GGCTCACTACCGTTGTCATCCT |  |  |  |

**Supplementary Table 2.** Antibiotic resistance phenotypes and genotypes of the *Enterobacteriaceae* isolates obtained from saliva samples obtained from healthy and chronic kidney disease patients.

|  |  | **Antibiotic resistance genes** | | | | | | **Mobile genetic elements** | | | | **Resistance phenotype** | | | | | | | | | | | | |
| --- | --- | --- | --- | --- | --- | --- | --- | --- | --- | --- | --- | --- | --- | --- | --- | --- | --- | --- | --- | --- | --- | --- | --- | --- |
|  |  | **β-lactamases** | | | | **PMQR*** | **Sulfonamides** | **Class 1 integrase** | **Plasmid replicons** | | | **Penicillins** | | **Cefalosporins** | | **Carbapenem** | **Colistin** | **Sulfonamides** | | **Quinolone** | **Tetracycline** | **Aminoglycosides** | |  |
| **Sample origin** | **Species** | ***bla*_CTX-M_** | ***bla*_OXA_** | ***bla*_SHV_** | ***bla*_TEM_** | ***qnrS*** | ***sul1*** | ***IntI1*** | **incF** | **incHI1** | **incHI2** | **AML** | **TIC** | **CP** | **CAZ** | **MEM** | **CT** | **SUL** | **SXT** | **CIP** | **TET** | **GEN** | **STR** |  |
| Healthy control | *Escherichia coli* | **-** | **-** | **-** | **-** | **-** | **+** | **-** | **-** | **-** | **-** | S | S | S | S | S | S | R | S | S | S | S | S |  |
| CKD patient | *Enterobacter asburiae*** | **-** | **-** | **-** | **-** | **-** | **+** | **+** | **-** | **-** | **-** | R | S | R | S | S | S | R | S | S | S | S | R |  |
| CKD patient | *Enterobacter asburiae*** | **-** | **-** | **-** | **-** | **-** | **-** | **-** | **-** | **-** | **-** | R | S | R | S | S | S | R | S | S | S | S | S |  |
| Healthy control | *Enterobacter asburiae* | **-** | **-** | **-** | **-** | **-** | **+** | **-** | **-** | **-** | **-** | S | S | R | S | S | S | R | S | S | S | S | S |  |
| Healthy control | *Klebsiella pneumoniae* | **-** | **-** | **+** | **-** | **-** | **+** | **-** | **-** | **-** | **-** | R | R | S | S | S | S | R | S | S | S | S | S |  |
| Healthy control | *Klebsiella pneumoniae* | **-** | **-** | **+** | **-** | **-** | **-** | **-** | **-** | **-** | **-** | R | R | S | S | S | S | R | S | S | S | S | S |  |
| CKD patient | *Klebsiella aerogenes* | **-** | **-** | **-** | **-** | **-** | **-** | **-** | **-** | **-** | **-** | R | S | R | S | S | S | S | S | S | S | S | S |  |
| CKD patient | *Klebsiella oxytoca* | **+** | **-** | **-** | **-** | **-** | **-** | **-** | **+** | **-** | **-** | R | S | S | S | S | S | S | S | S | S | S | S |  |
| CKD patient | *Citrobacter freundii* | **-** | **-** | **-** | **-** | **-** | **-** | **-** | **-** | **-** | **-** | R | S | S | S | S | S | S | S | S | S | S | S |  |
| CKD patient | *Raoultella ornithinolytica* | **-** | **-** | **-** | **-** | **-** | **-** | **-** | **-** | **-** | **-** | R | R | S | S | S | S | S | S | S | S | S | S |  |
| CKD patient | *Raoultella ornithinolytica* | **-** | **-** | **-** | **-** | **-** | **-** | **-** | **-** | **-** | **-** | R | R | S | S | S | S | S | S | S | S | S | S |  |
| CKD patient | *Raoultella ornithinolytica* | **-** | **-** | **-** | **-** | **-** | **-** | **-** | **-** | **-** | **-** | R | R | S | S | S | S | S | S | S | S | S | S |  |
| CKD patient | *Raoultella ornithinolytica*** | **-** | **-** | **-** | **-** | **-** | **+** | **+** | **-** | **-** | **-** | R | S | R | S | S | S | R | S | S | S | S | S |  |
| CKD patient | *Raoultella ornithinolytica*** | **-** | **-** | **-** | **-** | **-** | **+** | **+** | **-** | **-** | **-** | S | S | R | S | S | S | R | S | S | S | S | R |  |
| CKD patient | *Raoultella ornithinolytica*** | **-** | **-** | **-** | **-** | **-** | **+** | **+** | **-** | **-** | **-** | R | S | R | S | S | S | R | S | S | S | S | R |  |
| CKD patient | *Raoultella ornithinolytica* | **-** | **-** | **-** | **-** | **-** | **-** | **-** | **-** | **-** | **-** | R | R | S | S | S | S | S | S | S | S | S | S |  |
| CKD patient | *Raoultella ornithinolytica*** | **-** | **-** | **-** | **-** | **-** | **-** | **+** | **-** | **-** | **-** | S | S | R | S | S | S | R | S | S | S | S | R |  |
| CKD patient | *Raoultella ornithinolytica* | **-** | **-** | **-** | **-** | **-** | **-** | **-** | **-** | **-** | **-** | R | R | S | S | S | S | S | S | S | S | S | S |  |
| CKD patient | *Raoultella ornithinolytica* | **-** | **-** | **-** | **-** | **-** | **-** | **-** | **-** | **-** | **-** | R | R | S | S | S | S | S | S | S | S | S | S |  |
| CKD patient | *Raoultella ornithinolytica* | **-** | **-** | **-** | **-** | **-** | **-** | **-** | **-** | **-** | **-** | R | R | S | S | S | S | S | S | S | S | S | S |  |
| CKD patient | *Raoultella ornithinolytica* | **-** | **-** | **-** | **-** | **-** | **-** | **-** | **-** | **-** | **-** | R | R | S | S | S | S | S | S | S | S | S | S |  |
| CKD patient | *Raoultella ornithinolytica* | **-** | **-** | **-** | **-** | **-** | **-** | **-** | **-** | **-** | **-** | R | R | S | S | S | S | S | S | S | S | S | S |  |
| CKD patient | *Raoultella ornithinolytica* | **-** | **-** | **-** | **-** | **-** | **-** | **-** | **-** | **-** | **-** | R | R | S | S | S | S | S | S | S | S | S | S |  |

*PMQR: plasmid-mediated quinolone resistance

**Multidrug-resistant isolates, bacterial isolates resistant to at least one antibiotic in three or more distinct classes.

Antibiotics: AML, amoxicillin; TIC, ticarcillin; CP, cephalothin; CAZ, ceftazidime; MEM, meropenem; CT, colistin; SUL, sulfamethoxazole; SXT, sulfamethoxazole/trimethoprim; CIP, ciprofloxacin; TET, tetracycline; GEN, gentamycin; STR, streptomycin.

# Supplementary Figures


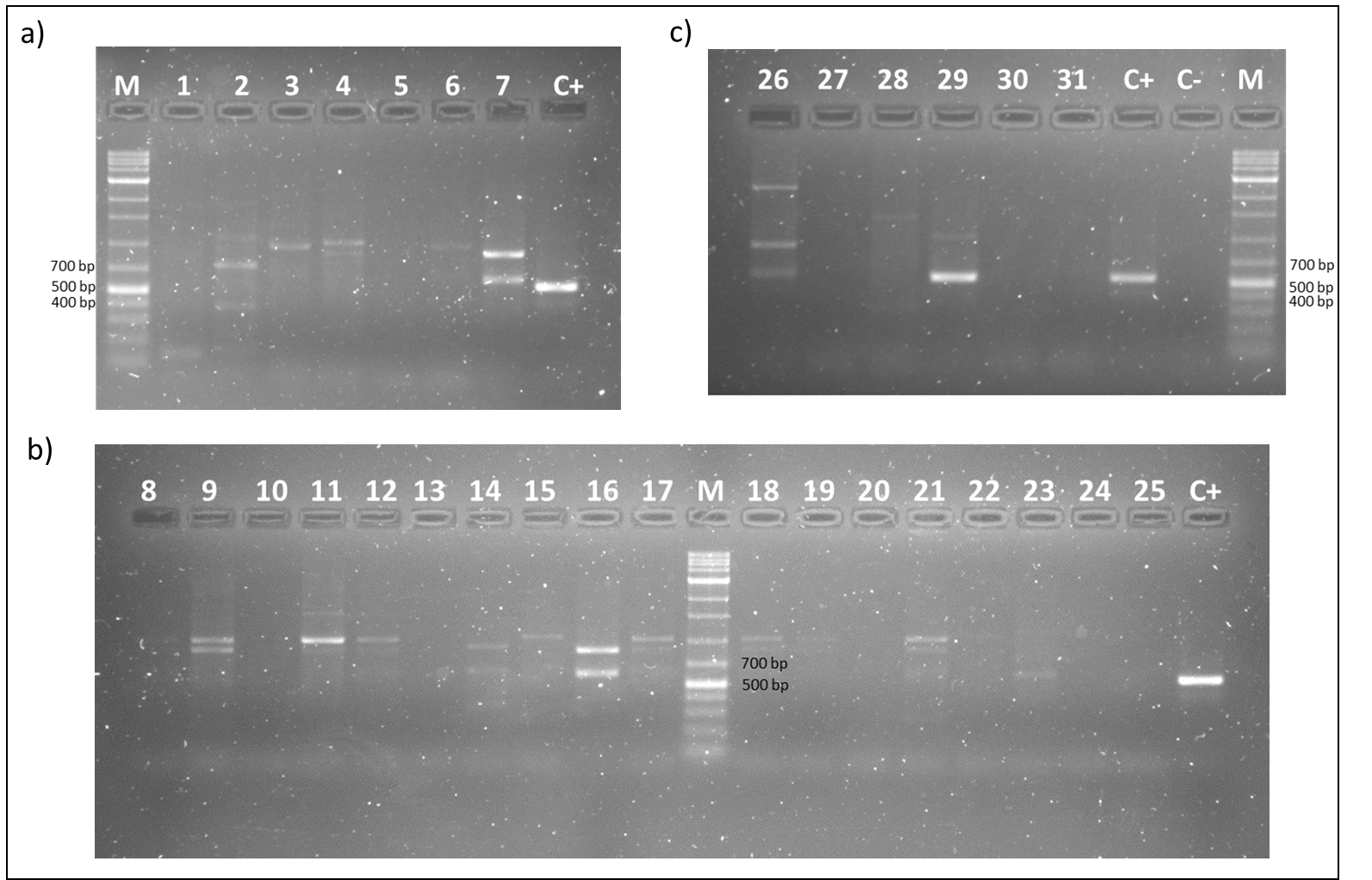


**Supplementary Figure 1.** Agarose gel electrophoresis of PCR amplification products of *bla*_CTX-M_ (540 bp) from all isolates (a, b, c). Isolates legend in the adjacent table. Irrelevant samples for the study were not included for analysis. Positive sample in lane 29. Gel 1.5% agarose, run time of 40 minutes. M, GRS Universal DNA ladder 10 000 bp (GRISP, Portugal); C+, positive control (*bla*_CTX-M_ +); C-, negative control (nuclease-free water).


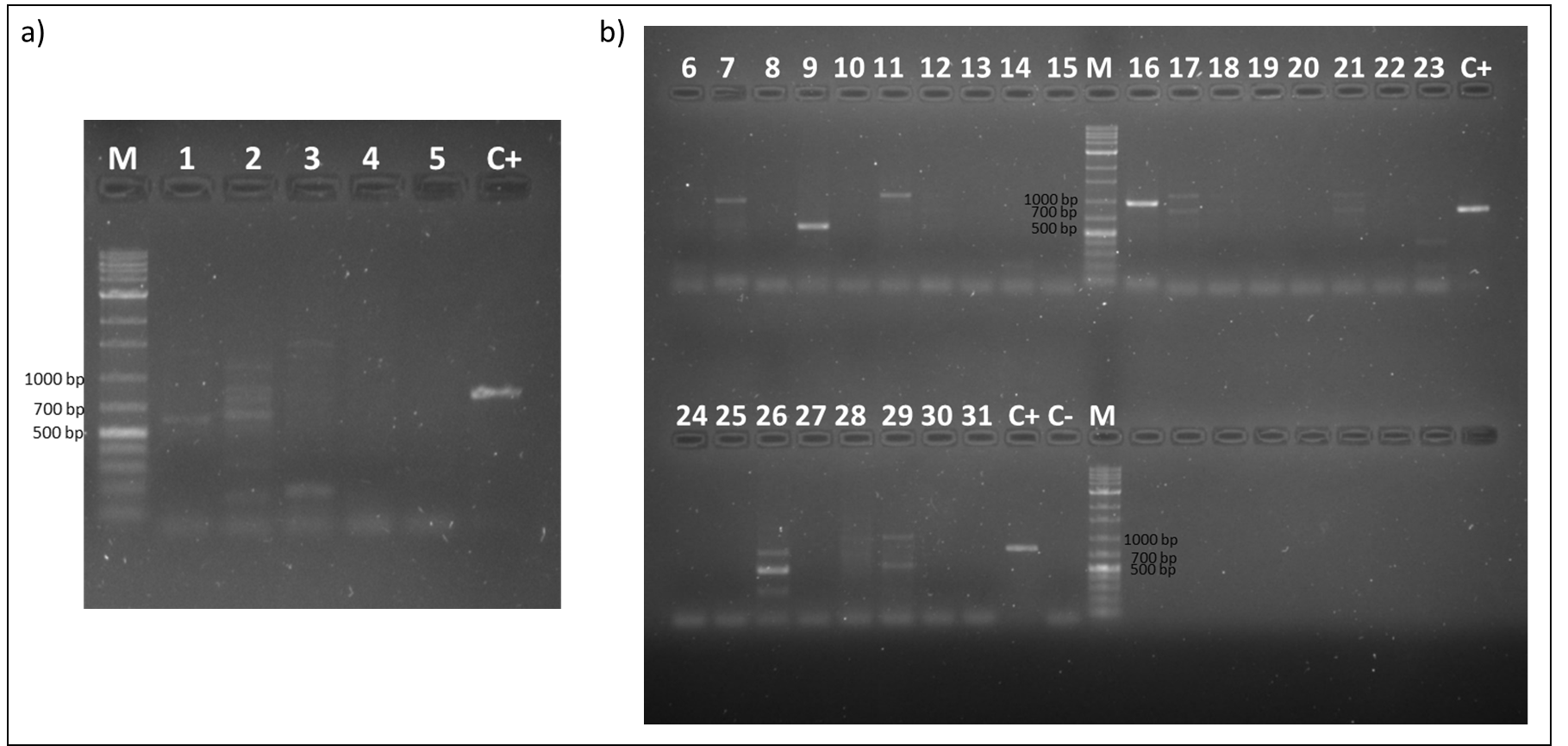


**Supplementary Figure 2.** Agarose gel electrophoresis of PCR amplification products of *bla*_OXA-A_ (840 bp) from all isolates (a, b). Isolates legend in the adjacent table. Irrelevant samples for the study were not included for analysis. Gel 1.5% agarose, run time of 55 minutes. M, GRS Universal DNA ladder 10 000 bp (GRISP, Portugal); C+, positive control (*bla*_OXA-A_ +); C-, negative control (nuclease-free water).


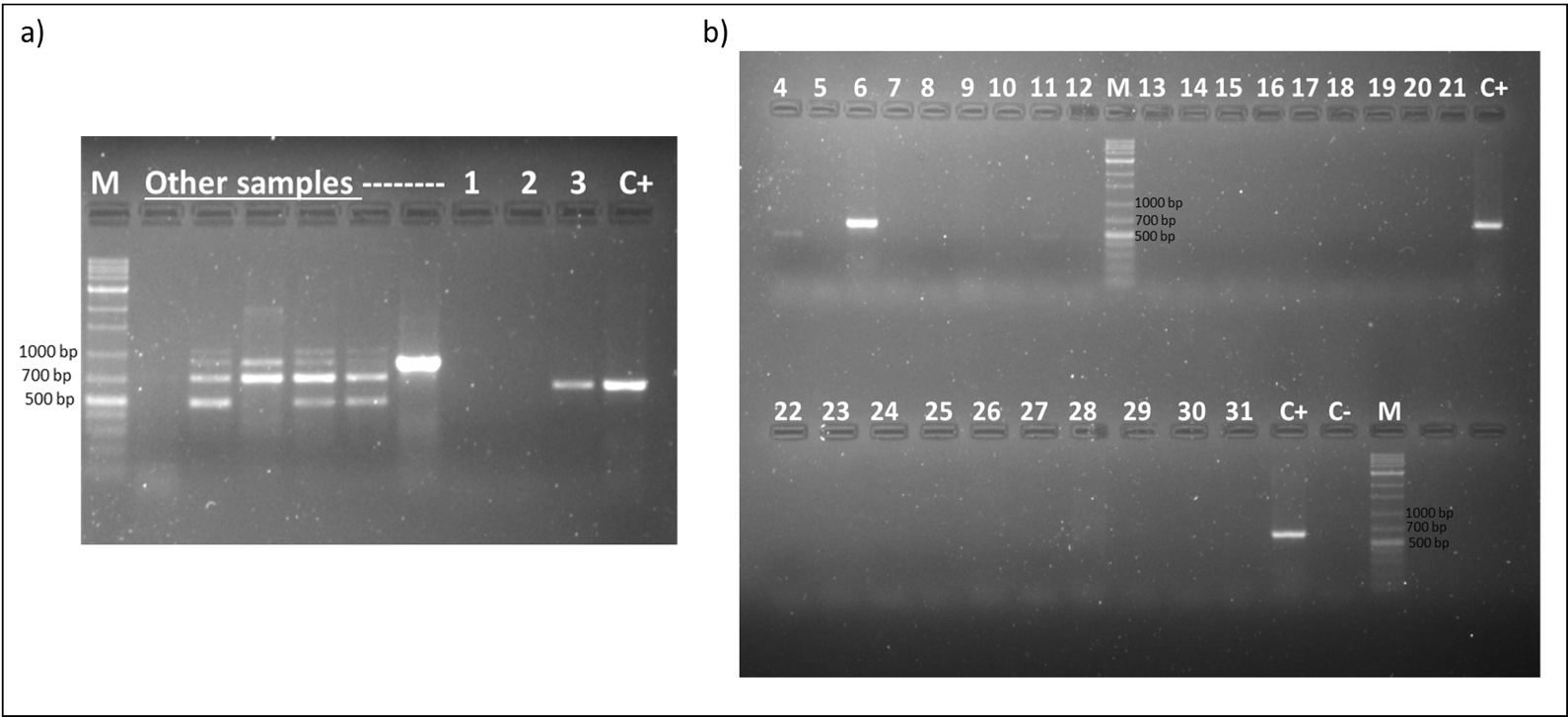

**Supplementary Figure 3.** Agarose gel electrophoresis of PCR amplification products of blaSHV (626 bp) from all isolates (a, b). Isolates legend in the adjacent table. Irrelevant samples for the study were not included for analysis. Positive samples in lanes 3 and 6. Gel 1.5% agarose, run time of 40 minutes. M, GRS Universal DNA ladder 10 000 bp (GRISP, Portugal); C+, positive control (*bla*_SHV_ +); C-, negative control (nuclease-free water).


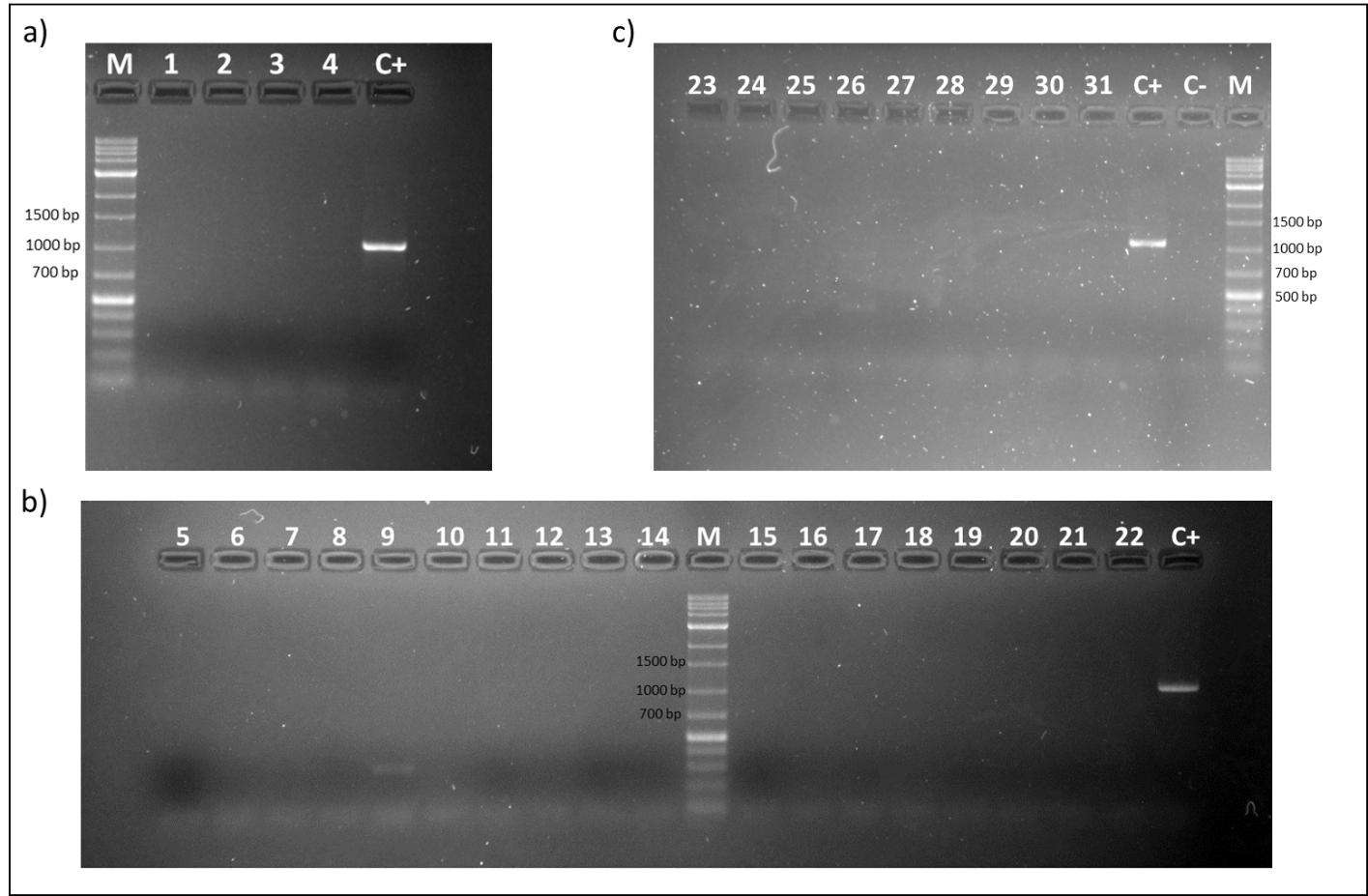

**Supplementary Figure 4.** Agarose gel electrophoresis of PCR amplification products of *bla*_TEM_ (1090 bp) from all isolates (a, b, c). Isolates legend in the adjacent table. Irrelevant samples for the study were not included for analysis. Gel 1.5% agarose, run time of 40 minutes. M, GRS Universal DNA ladder 10 000 bp (GRISP, Portugal); C+, positive control (*bla*_TEM_ +); C-, negative control (nuclease-free water).


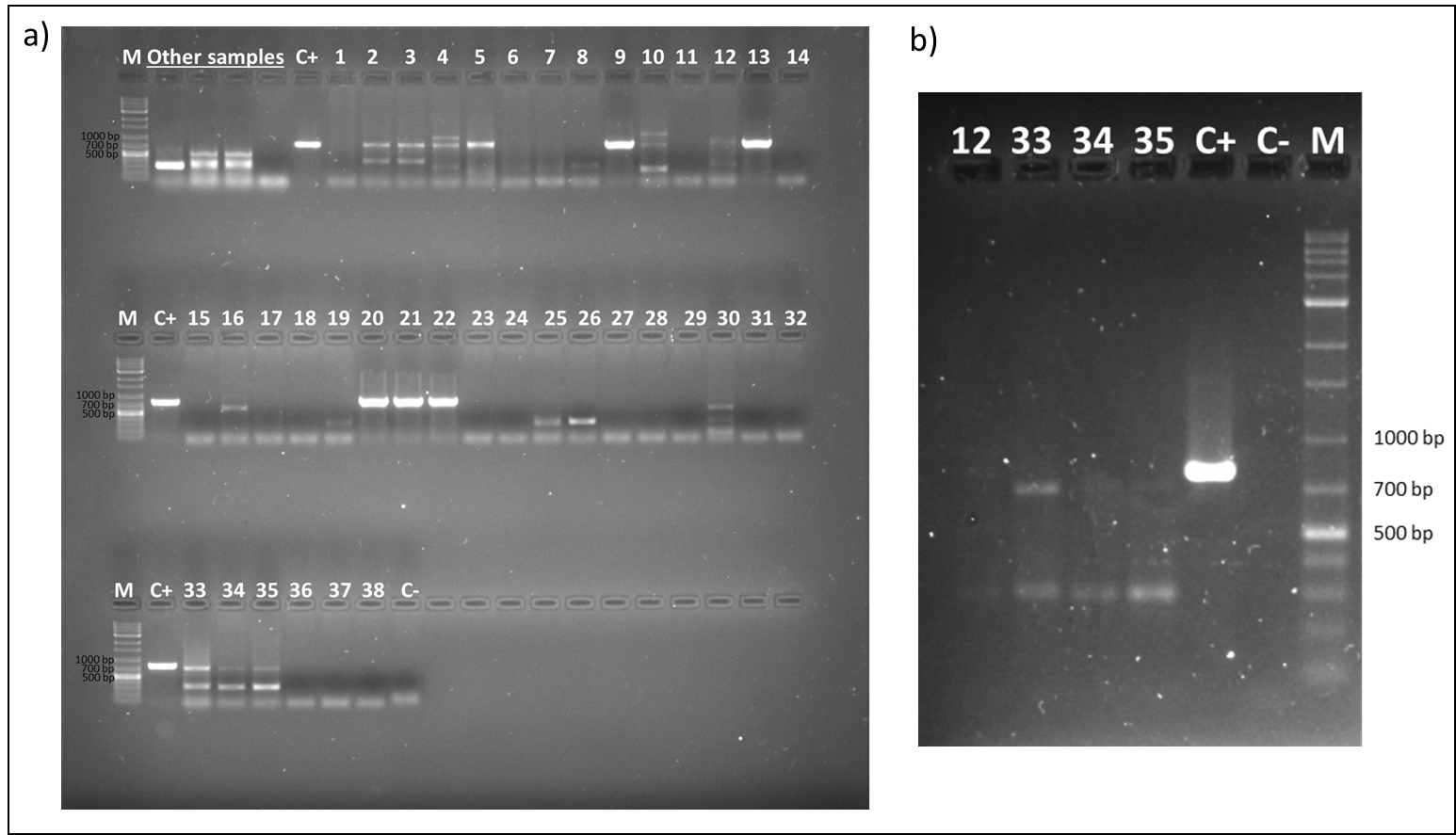


**Supplementary Figure 5.** a) Agarose gel electrophoresis of PCR amplification products of *sul1* (789 bp) from all isolates (a; b – replicas for some reactions that were difficult to observe in gel (a)). Isolates legend in the adjacent table. Irrelevant samples for the study were not included for analysis. Positive samples in lanes 2, 4, 5, 9, 20-22. Gel 1.5% agarose, run time of 30 minutes (60 minutes in the repetition). M, GRS Universal DNA ladder 10 000 bp (GRISP, Portugal); C+, positive control (*sul1* +); C-, negative control (nuclease-free water).


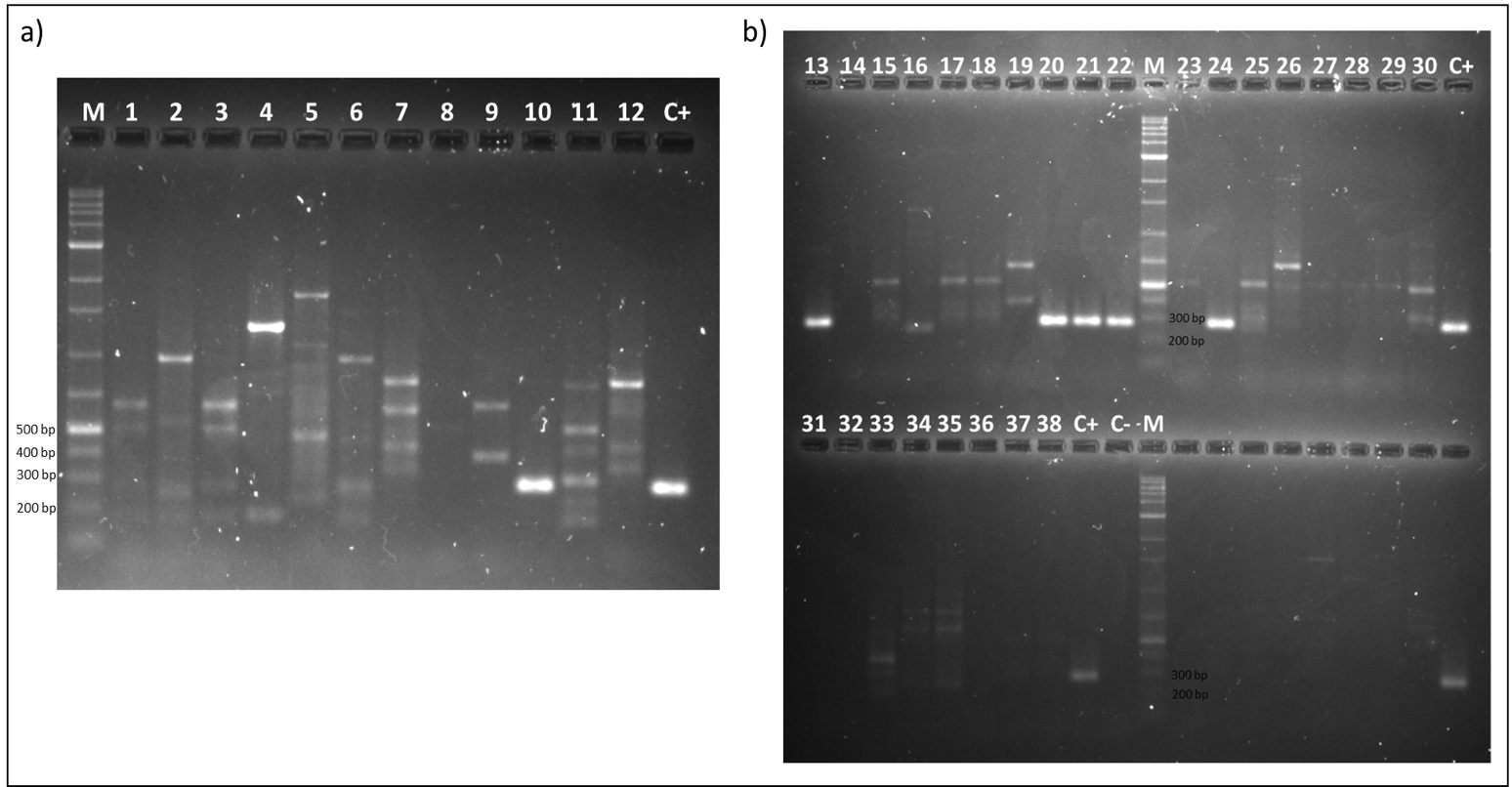

**Supplementary Figure 6.** Agarose gel electrophoresis of PCR amplification products of *intI1* (280 bp) from all isolates (a, b). Isolates legend in the adjacent table. Irrelevant samples for the study were not included for analysis. Positive samples in lanes 10, 20-22, 24. Gel 1.5% agarose, run time of 50 minutes. M, GRS Universal DNA ladder 10 000 bp (GRISP, Portugal); C+, positive control (*intI1* +); C-, negative control (nuclease-free water).


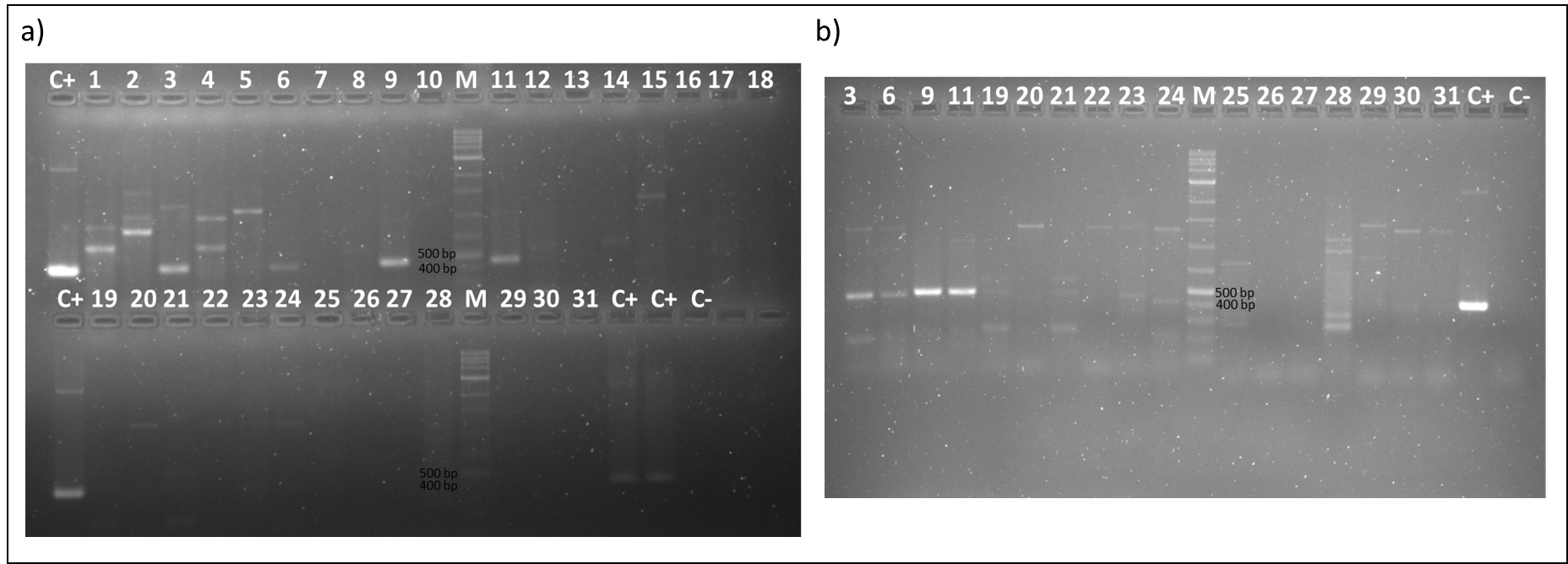


**Supplementary Figure 7.** a) Agarose gel electrophoresis of PCR amplification products of *qnrS* (428 bp) from all isolates (a; b – replicas for some reactions that were difficult to observe in the gel (a)). Isolates legend in the adjacent table. Irrelevant samples for the study were not included for analysis. Gel 1.5% agarose, run time of 40 minutes (60 minutes in the repetition). M, GRS Universal DNA ladder 10 000 bp (GRISP, Portugal); C+, positive control (*qnrS* +); C-, negative control (nuclease-free water).


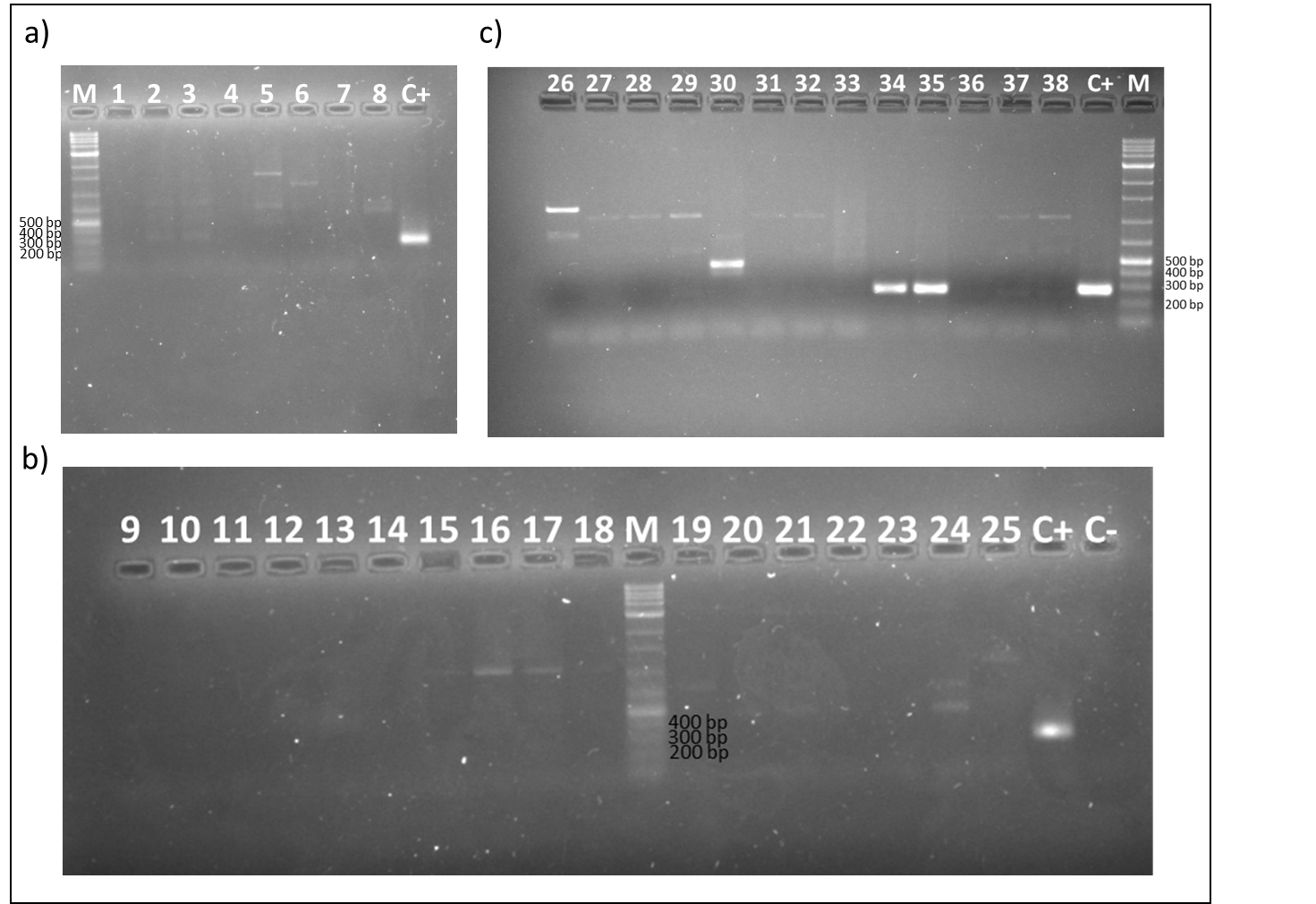

**Supplementary Figure 8.** Agarose gel electrophoresis of PCR amplification products for incF (270 bp) from all isolates (a, b, c). Isolates legend in the adjacent table. Irrelevant samples for the study were not included for analysis. Positive sample in lane 34. Gel 1.5% agarose, run time of 40 minutes. M, GRS Universal DNA ladder 10 000 bp (GRISP, Portugal); C+, positive control (incF +); C-, negative control (nuclease-free water).


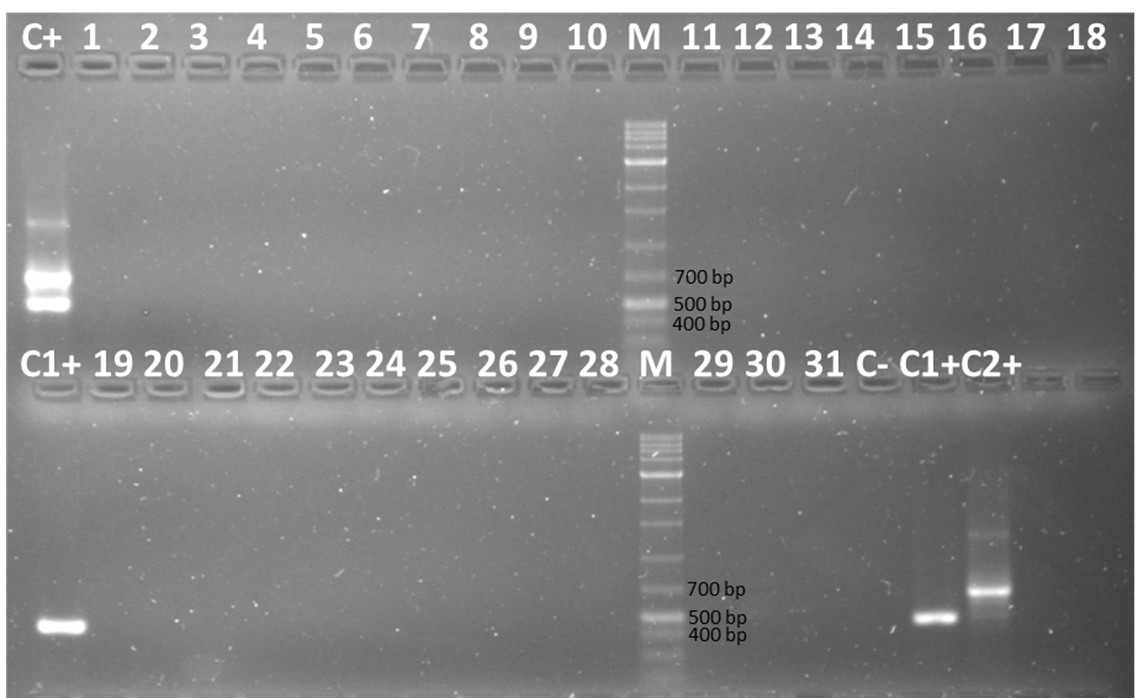


**Supplementary Figure 9.** Agarose gel electrophoresis of PCR amplification products for HI1 (471 bp) and HI2 (644 bp) from all isolates. Isolates legend in the adjacent table. Irrelevant samples for the study were not included for analysis. Gel 1.5% agarose, run time of 60 minutes. M, GRS Universal DNA ladder 10 000 bp (GRISP, Portugal); C+, positive control for both HI1 and HI2 (HI1 + and HI2 +); C1+, positive control for HI1 (HI1 +); C2+, positive control for HI2 (HI2 +); C-, negative control (nuclease-free water).

#
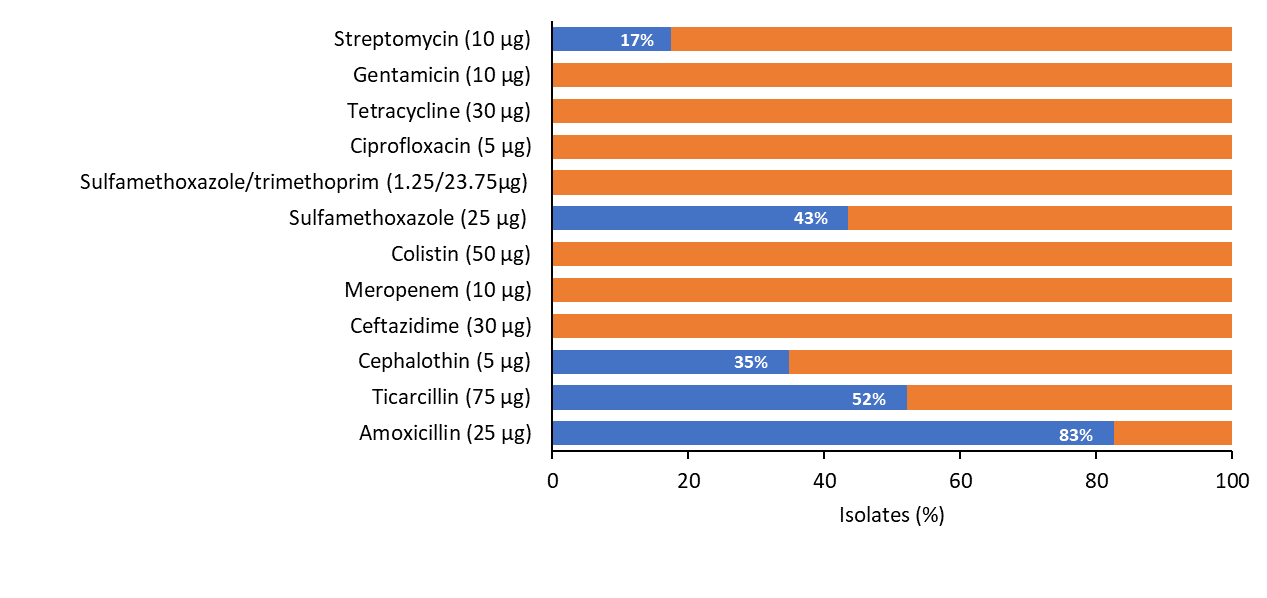


**Supplementary Figure 10.** Antibiotic susceptibility pattern of all isolates (n=23) for the twelve antibiotics tested. ■ Resistant, ■ Susceptible.

**
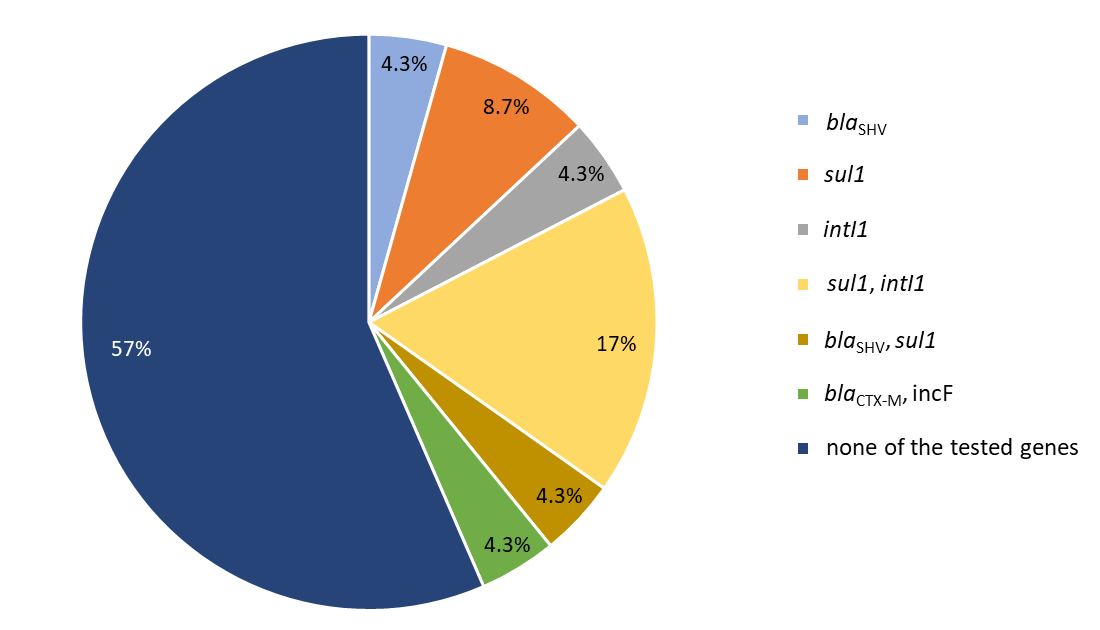
**

**Supplementary Figure 11.** Prevalence (%) of detected antibiotic-resistance genes (*bla*_CTX-M_, *bla*_SHV_, and *sul1*), class I integron integrase (*intI1*) and plasmid replicon type (incF) in the isolates (n=23). The antibiotic resistance genes *bla*_OXA-A_ *bla*_TEM_, *qnrS,* and plasmid replicon types incHI1 and incHI2, were not detected in the isolates.

# Supplementary References

Carattoli, A., Bertini, A., Villa, L., Falbo, V., Hopkins, K. L., and Threlfall, E. J. (2005). Identification of plasmids by PCR-based replicon typing. *J. Microbiol. Methods*. 63, 219–228.

Cattoir, V., Poirel, L., Rotimi, V., Soussy, C. J., and Nordmann, P. (2007). Multiplex PCR for detection of plasmid-mediated quinolone resistance qnr genes in ESBL-producing enterobacterial isolates. *J. Antimicrob. Chemother.* 60, 394–397.

DiPersio, J. R., Deshpande, L. M., Biedenbach, D. J., Toleman, M. A., Walsh, T. R., and Jones, R. N. (2005). Evolution and dissemination of extended-spectrum β-lactamase-producing *Klebsiella pneumoniae*: Epidemiology and molecular report from the SENTRY Antimicrobial Surveillance Program (1997-2003). *Diagn. Microbiol. Infect. Dis*. 51, 1–7.

Henriques, I. S., Fonseca, F., Alves, A., Saavedra, M. J., and Correia, A. (2006). Occurrence and diversity of integrons and β-lactamase genes among ampicillin-resistant isolates from estuarine waters. *Res. Microbiol*. 157, 938–947.

Reis, P. J. M., Homem, V., Alves, A., Vilar, V. J. P., Manaia, C. M., and Nunes, O. C. (2018). Insights on sulfamethoxazole bio-transformation by environmental Proteobacteria isolates. *J. Hazard. Mater*. 358, 310–318.

Weill, F. X., Lailler, R., Praud, K., Kérouanton, A., Fabre, L., Brisabois, A., Grimont, P. A. D., and Cloeckaert, A. (2004). Emergence of extended-spectrum-β-lactamase (CTX-M-9)-producing multiresistant strains of *Salmonella enterica* serotype virchow in poultry and humans in France. *J. Clin. Microbiol*. 42, 5767–5773.
